# *Vibrio* Type III Effector VPA1380 Is Related to the Cysteine Protease Domain of Large Bacterial Toxins

**DOI:** 10.1371/journal.pone.0104387

**Published:** 2014-08-06

**Authors:** Thomas Calder, Lisa N. Kinch, Jessie Fernandez, Dor Salomon, Nick V. Grishin, Kim Orth

**Affiliations:** 1 Department of Molecular Biology, University of Texas Southwestern Medical Center, Dallas, Texas, United States of America; 2 Department of Biophysics and Biochemistry, University of Texas Southwestern Medical Center, Dallas, Texas, United States of America; 3 Howard Hughes Medical Institute, University of Texas Southwestern Medical Center, Dallas, Texas, United States of America; Centre National de la Recherche Scientifique, Aix-Marseille Université, France

## Abstract

*Vibrio parahaemolyticus* is a Gram-negative halophilic bacterium and one of the leading causes of food-borne gastroenteritis. Its genome harbors two Type III Secretion Systems (T3SS1 and T3SS2), but only T3SS2 is required for enterotoxicity seen in animal models. Effector proteins secreted from T3SS2 have been previously shown to promote colonization of the intestinal epithelium, invasion of host cells, and destruction of the epithelial monolayer. In this study, we identify VPA1380, a T3SS2 effector protein that is toxic when expressed in yeast. Bioinformatic analyses revealed that VPA1380 is highly similar to the inositol hexakisphosphate (IP6)-inducible cysteine protease domains of several large bacterial toxins. Mutations in conserved catalytic residues and residues in the putative IP6-binding pocket abolished toxicity in yeast. Furthermore, VPA1380 was not toxic in IP6 deficient yeast cells. Therefore, our findings suggest that VPA1380 is a cysteine protease that requires IP6 as an activator.

## Introduction

*Vibrio parahaemolyticus* is a Gram-negative bacterium and one of the major etiological agents of acute gastroenteritis derived from eating raw or undercooked shellfish [1]. Disease symptoms include abdominal cramps, headache, nausea, vomiting, and mild to severe diarrhea [2]. If exposed to open wounds, the bacterium can cause wound infections and even septicemia [3]. In recent decades, *V. parahaemolyticus* has emerged as an important pathogen due to its dissemination to coastal regions throughout the world [4]. The bacterium’s preference for warm temperatures and the rising of global water temperatures appear to be aiding this spread [5].

The genome sequence of the *V. parahaemolyticus* RIMD 2210633 clinical isolate revealed several virulence factors in pathogenicity islands [6]. These pathogenicity islands include two type III secretion systems (T3SS), T3SS1 and T3SS2 [7], and two type VI secretion systems (T6SS), T6SS1 and T6SS2 [8]. Also within the T3SS2 pathogenicity island, are genes for two thermodirect-hemolysin (*tdh*) toxins. Importantly, the *V. parahaemolyticus* T3SS2 is induced by bile salts [9] and was shown to be required for intestinal colonization and enterotoxicity, as shown in several infection model organisms [7], [10]–[12], albeit septicemia has yet to be observed in an animal model. In culture systems, the T3SS2 system was shown to mediate invasion [13]. The T3SS is a needle-like apparatus that delivers bacterial effector proteins into the host cell cytoplasm. Once inside the host cell, effector proteins target different host machinery to manipulate the immune response and to promote a niche outside or within the host cell [14]. Six T3SS2 effector proteins have been identified and characterized to-date. VopA and VopZ both target cellular immunity by inhibiting different components of the MAPK pathway [12], [15]. VopT ADP-ribosylates Ras, and the three other effectors, VopC, VopL, and VopV, alter actin dynamics [13], [16], [17]. Notably, VopC constitutively activates Cdc42 through a deamidation reaction to promote invasion of the bacterium into non-phagocytic cells [13], [18]. This data suggests bacterial invasion may play a role during infection.

Previous work on T3SS2 in *V. parahaemolyticus* identified the protein VPA1380 as a T3SS2-dependent secreted protein [17]. In this work, we set out to determine whether VPA1380 is a T3SS2 effector and characterize its activity. To this end, we confirmed that VPA1380 is translocated into host cells by T3SS2. Bioinformatic analyses revealed that VPA1380 is similar to cysteine protease domains (CPDs) from several large bacterial toxins. Using yeast as a heterologous model, we found that VPA1380’s toxicity is abolished when the putative catalytic residues of the protease domain are mutated. Furthermore, we show that like other CPDs found in bacterial toxins, VPA1380 requires IP6 as an activator. Thus, our results suggest that VPA1380 is a T3SS2 effector with an IP6-dependent catalytic activity.

## Results

### VPA1380 is a T3SS2 Effector

**Figure 1:**
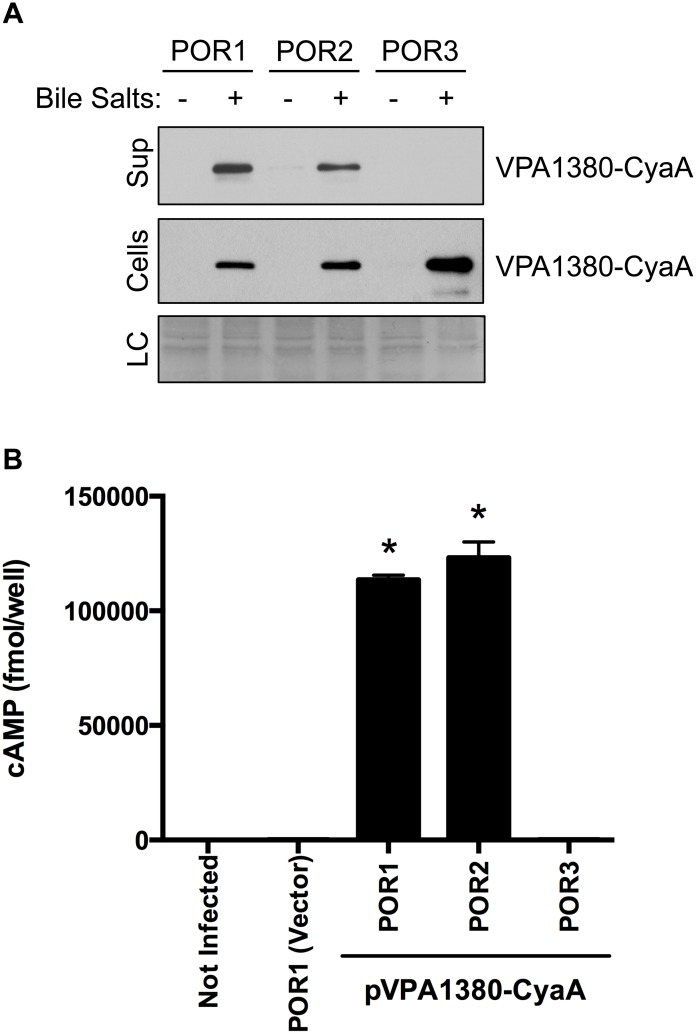
VPA1380 is a T3SS2 effector. (**A**) Secretion of VPA1380-CyaA from *Vibrio parahaemolyticus* strains POR1(T3SS1+/T3SS2+), POR2(T3SS1−/T3SS2+), and POR3(T3SS1+/T3SS2–) as detected by immunoblot analysis from bacterial cell lysate or TCA-precipitated media. Blots were probed with anti-CyaA antibody. Loading control (LC) is shown for total protein lysate. (**B**) Measurement of translocated VPA1380-CyaA after infection of HeLa cells with *V. parahaemolyticus* strains for 1 hour. Intracellular cAMP levels were quantified by ELISA system. Asterisks indicate statistical significant differences between POR1(VPA1380-CyaA) and POR3(VPA1380-CyaA) (*P* = 0.0001, *n* = 3) and between POR2(VPA1380-CyaA) and POR3(VPA1380-CyaA) (*P* = 0.001, *n* = 3), using two-tailed *t*-test.

While examining the genes within the T3SS2 pathogenicity island (*vpa1310*–*vpa1396*) we identified *vpa1380* as a putative T3SS2 effector. The *vpa1380* gene encodes for a 295 amino acid protein, which is homologous to the partially characterized OspB effector from *Shigella flexneri* (32% identity and 50% similarity) [19], [20] (Fig. S1). VPA1380 was recently shown to be secreted from *V. parahaemolyticus* via the T3SS2 [17]. To confirm VPA1380 is secreted from T3SS2, we transformed *V. parahaemolyticus* strains with a plasmid containing *vpa1380* fused to the catalytic region of the *cyaA* gene with *vpa1380’s* endogenous promoter. The POR1 strain contains a functional T3SS1 and T3SS2, while the POR2(POR1Δ*vcrD1*) and POR3(POR1Δ*vcrD2*) strains harbor a mutation in the needle apparatus of T3SS1 and T3SS2, respectively [21]. Upon induction of T3SS2 with bile salts [9], we found VPA1380-CyaA was produced in all tested strains but only secreted from strains containing an active T3SS2 (POR1(T3SS1+/T3SS2+) and POR2(T3SS1−/T3SS2+)), and not from a strain with an inactive T3SS2 (POR3(T3SS1+/T3SS2–)) (Fig. 1A). These results confirm that VPA1380 is secreted through the T3SS2.

To determine if VPA1380 is *a bona fide* T3SS2 effector, we next determined whether VPA1380 is translocated into eukaryotic host cells in a T3SS2-dependent manner using the CyaA reporter system. *V. parahaemolyticus* strains expressing the VPA1380-CyaA fusion were used to infect HeLa cells, and production of cAMP was monitored. HeLa cells infected with POR1(T3SS1+/T3SS2+) and POR2(T3SS1−/T3SS2+) exhibited significantly higher levels of cAMP than cells infected with POR3(T3SS1+/T3SS2–) (Fig. 1B). Taken together, these results confirmed that VPA1380 is indeed a translocated T3SS2 effector.

### VPA1380 is detrimental when expressed in yeast

**Figure 2:**
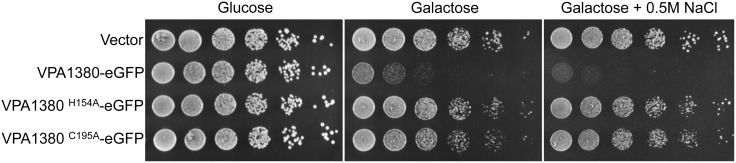
VPA1380 is toxic in yeast. Growth of yeast expressing VPA1380-eGFP and putative active site mutants. 5-fold serial dilutions of yeast were spotted on repressing (glucose) or inducing (galactose) medium. Additionally, yeast were spotted on inducing medium with osmotic stress (0.5 M NaCl M NaCl).

To determine whether VPA1380 targets a conserved eukaryotic process, we used yeast as a eukaryotic heterologous system. Yeast are commonly used to study T3SS effectors since the targets of T3SS effectors are often conserved across eukaryotic species [22]–[24]. VPA1380 inhibited yeast growth when expressed as an eGFP-fusion from a galactose-inducible promoter (Fig. 2). Previous studies of bacterial T3SS effectors in yeast showed that effector–mediated toxicity can be revealed or exacerbated in the presence of stress conditions [22], [23]. Interestingly, VPA1380-mediated yeast growth inhibition was exacerbated in the presence of osmotic stress by addition of 0.5 M NaCl to the medium (Fig. 2). These results suggest that VPA1380 targets a conserved eukaryotic process found in yeast.

### VPA1380 is similar to the cysteine protease domain of other bacterial toxins

**Figure 3:**
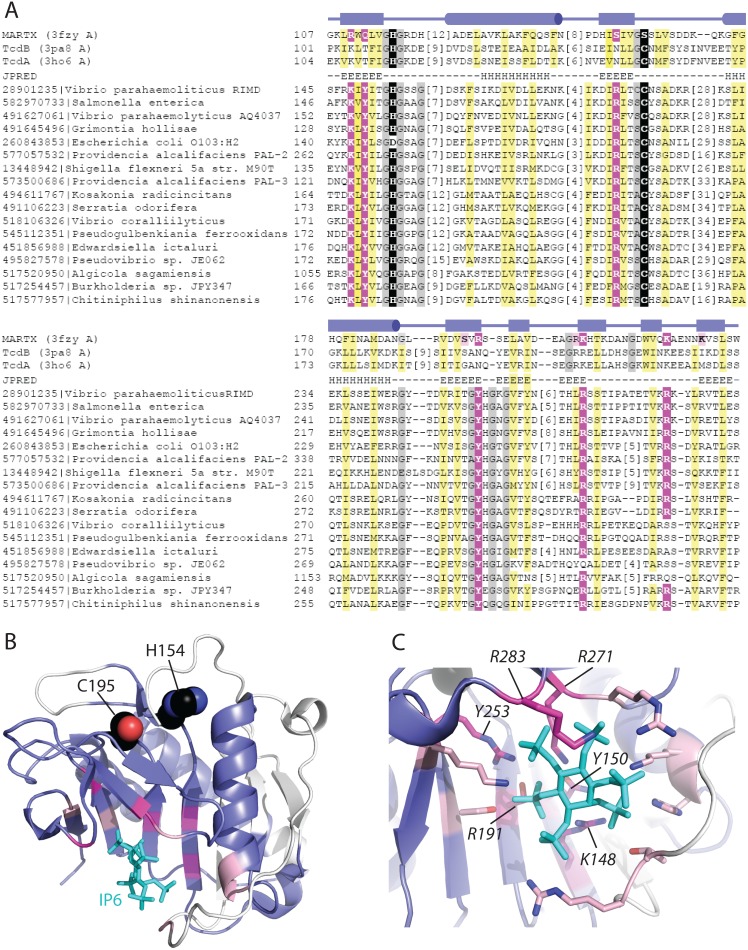
VPA1380 is related to the IP6-activated cysteine protease domain of MARTX and CGT toxins. (**A**) Multiple sequence alignment (missing first mapped helix) of IP6-binding MARTX and CGT toxin CPD structures (top, labeled to the left according to gene name and PDB ID) with representative VPA1380-related sequences (bottom, labeled to the left according to NCBI GI and species) highlights conserved active site (black highlights) and IP6 binding residues (magenta and pink highlighted). The observed MARTX secondary structures (boxes for strands and cylinders for helices above the alignment) correspond to the VPA1380 predicted secondary structures (E for strands and H for helices), and the hydrophobicity patterns match (mainly hydrophobic positions highlighted yellow, and mainly small positions highlighted gray). (**B**) A ribbon representation of the *Vibrio cholerae* MARTX cysteine protease domain (PDB 3fxy) depicts the region of the structure that confidently aligns to VPA1380 (colored slate, with unaligned region in white). The aligned region encompasses the Cys-His active site (black spheres), and a majority of the IP6 (cyan stick) binding site (magenta and pink). (**C**) A zoom of the IP6 binding site highlights MARTX residues that form hydrogen-bonds to IP6 and map to conserved VPA1380 positions (magenta sticks, labeled in italics according to VPA1380 residues). Several MARTX residues that form the IP6 binding pocket were either mapped to the unaligned N-terminus or were not mutated (pink sticks).

Despite the fact that VPA1380 homologs from various bacteria have been previously identified as T3SS effectors, their mechanism of action remains unknown. To shed light on the molecular function of VPA1380, we sought to identify related structures using a sensitive sequence detection method (HHPRED). Top hits to the VPA1380 sequence query include the CPD of multifunctional-autoprocessing RTX (MARTX) from *Vibrio cholerae* and clostridial glucosylating toxins (CGTs), TcdB and TcdA, from *Clostridium difficile* (MARTX (3fzy A) with probability 97.9%, TcdB (3pa8 A) with probability 95.8%, and TcdA (3ho6 A) with probability 95.3%) (Fig. 3A).

These large bacterial toxins contain a CPD that requires the eukaryotic signaling molecule inositol hexakisphosphate (IP6) for activation. Studies to understand this process have been mainly focused on the CPD from the *V. cholerae* MARTX toxin and the *C. difficile* CGT toxins, TcdA and TcdB [25]–[27]. These multi-domain toxins gain access to the host cytosol by forming a pore in the host membrane, which allows their CPD to bind IP6 and initiate autoprocessing of the toxin. This autoprocessing event allows for the release of toxin effector domains, which function to alter different cellular processes [28]. The CPDs from MARTX and CGT toxins contain a catalytic dyad with a conserved histidine and cysteine, along with a conserved IP6 binding pocket comprised mostly of positive charged residues. IP6 is thought to activate the CPD by initiating conformational folding of the catalytic site [26], [29].

Resulting HHPRED alignments map to the C-terminal region of VPA1380 (residues 116–268) and encompass the conserved Cys-His active site residues (Fig. 3A, B). Interestingly, several conserved VPA1380 positions map to residues that mediate binding to IP6 (Fig. 3A, C), including several invariant residues (K148, Y150, R191 and Y253) and several conserved residues (R271 and R283) that are capable of forming similar interactions (Fig. 3C).

While VPA1380 retains all of the active site residues common to the CPDs of MARTX and CGT toxins, the sequence diverges at the N-terminus (Figure 3 alignment starts with residue 145). In fact, the N-terminal portion of the VPA1380 CPD more closely resembles a more distantly related cysteine protease (VPA1380 residues 52–202 maps to *Caenorhabditis elegans* 4m9r with HHPRED probability 75.34%). This relationship extends the coverage of the VPA1380 sequence to include the complete CPD fold. We found VPA1380Δ50-eGFP, lacking the far N-terminal region (aa 1–50), still reduced yeast growth (Fig. S2), which suggests only the CPD region is sufficient for VPA1380’s toxicity in yeast.

### Putative active site residues are required for VPA1380’s toxicity in yeast

To examine whether the putative Cys-His catalytic dyad that we identified is required for VPA1380’s activity, we examined the effects of corresponding point mutations on VPA1380’s toxicity in yeast. Whereas galactose-inducible expression of VPA1380-eGFP greatly reduced yeast growth, the toxic effects were abolished when the putative active site residues (H154 and C195) were mutated to an alanine (Fig. 2). Moreover, no growth inhibition was observed when VPA1380^H154A^-eGFP and VPA1380^C195A^-eGFP were expressed in yeast in the presence of osmotic stress (Fig. 2). Importantly, all of the mutated forms of VPA1380 and the wild-type effector were expressed (Fig. S3). Therefore, these conserved residues appear to be critical for VPA1380’s toxicity.

### VPA1380’s toxicity is dependent on IP6 and putative IP6 binding residues

**Figure 4:**
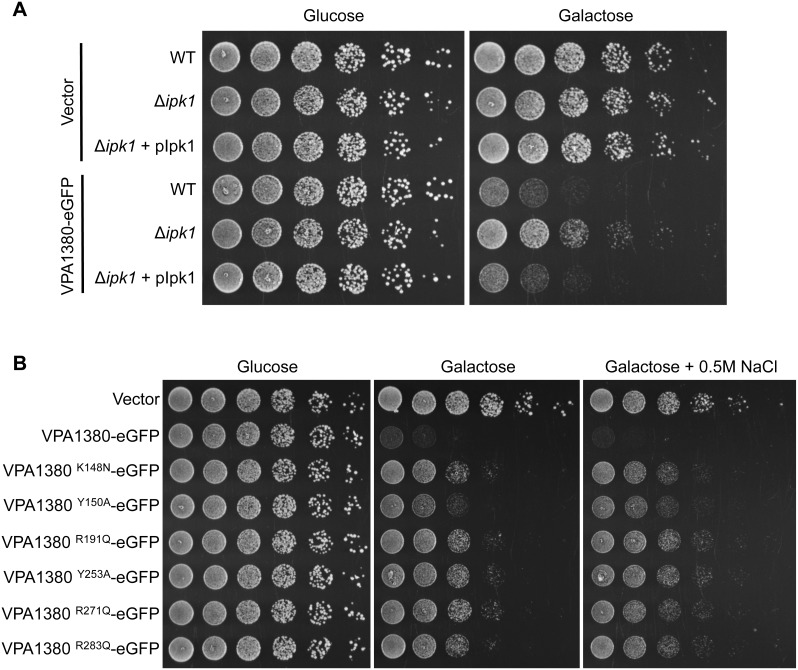
VPA1380 requires IP6 and putative IP6 binding residues for toxicity in yeast. (**A**) Growth of yeast strains: wt, Δ*ipk1* IP6-deficient, and Δ*ipk1*+ pIpk1 complemented yeast, expressing eGFP or VPA1380-eGFP. 5-fold serial dilutions of yeast were spotted on repressing (glucose) or inducing (galactose) plates. (**B**) Growth of Yeast expressing VPA1380-eGFP or putative IP6-binding mutants. Additionally, yeast were grown on inducing plates with osmotic stress (0.5 M NaCl M NaCl).

As the cysteine proteases similar to VPA1380 are known to use IP6 as an activator, we next set out to determine whether VPA1380 requires IP6 to cause yeast growth inhibition. In yeast, the kinase Ipk1 is responsible for converting IP5 to IP6 [30]. The *ipk1* yeast deletion strain of *Saccharomyces cerevisiae* is unable to make IP6 and yet is still viable, which allowed us to examine the IP6-dependency of VPA1380. Interestingly, deletion of *ipk1* greatly suppressed the VPA1380-mediated yeast growth inhibition. The inhibition was restored when expression of Ipk1 was complemented from a plasmid in the *ipk1* deletion strain (Fig. 4A). Importantly, VPA1380-eGFP was expressed in the Δ*ipk1* strain and the reconstituted strain (Fig. S4A). These results suggest that IP6 is required for the VPA1380-mediated toxicity in yeast.

Since IP6 appeared to be required for the activity of VPA1380, we next sought to verify the importance of the six putative IP6-binding residues, which we identified in VPA1380 (Fig. 3A, C). The positively charged residues (K148, R191, R271, and R283) were mutated to similar sized residues, asparagine and glutamine, respectively, without a charge to mitigate the effects on protein structure, while still removing the specific hydrogen-bonding ability of the residues. Additionally, the tyrosine residues (Y150 and Y253) were mutated to an alanine to also eliminate possible hydrogen-bonding interactions. All six point mutants expressed at a similar level (Fig. S4B) and were significantly less toxic when expressed in yeast, though the Y150A mutation appeared to have an intermediate effect (Fig. 4B). To better visualize the difference in yeast inhibition, we also tested the effect of these mutants in the presence of osmotic stress (0.5 M NaCl), which exacerbates the VPA1380-mediated growth inhibition (Fig. 2). Indeed, the toxicity differences described above were more apparent under the stress condition (Fig. 4B). Taken together, the dependence of VPA1380 on IP6 and the six conserved residues that contribute to the putative IP6 binding pocket in VPA1380, support our hypothesis that IP6 is likely required for VPA1380’s activity.

## Discussion

In this work, we identified and characterized a new *V. parahaemolyticus* T3SS2 effector, VPA1380. From bioinformatic analyses, we identified the putative catalytic and IP6-binding residues that are critical for VPA1380’s activity in yeast. This supports the comparison of VPA1380 with the CPDs of MARTX and CGT toxins since these toxins also require the conserved catalytic and IP6-binding residues for enzyme activity [25], [26], [31], [32]. Unlike the autoprocessing MARTX and CGT toxins, VPA1380 did not appear to cleave itself since no cleavage fragments were observed (Fig. S3). Therefore, VPA1380 is likely a cysteine protease that requires IP6 as an activator and targets a host substrate.

Many T3SS effectors require a eukaryotic factor for their activity. This regulatory control is thought to prevent off target effects prior to translocation that may arise from a promiscuous enzyme [33]. YpkA is an effector from *Yersinia* that requires actin from a host cell for activation of its kinase domain [34]. The OspG effector from *Shigella* binds to the host protein ubiquitin in conjugated, chained or free form to turn on its kinase activity [35]. AvrRpt2 is a T3SS effector from *Pseudomonas syringae* that binds to the protein cyclophilin in *Arabidopsis* plant cells to activate its protease activity [36]. Cyclophilin was found to mediate structural folding of AvrRpt2 through its binding and peptidyl-prolyl isomerase activity [37]. These effectors are representative examples that serve to exemplify how effector-activation by a eukaryotic factor is a strategy widely used by different T3SS effectors from numerous pathogenic bacteria.

VPA1380 is homologous to two partially characterized T3SS effector proteins. A homolog from pathogenic *E. coli* strains, named Ibe, was demonstrated through pull down experiments and localization studies to interact with the scaffolding protein IQGAP1 at the bacterial-induced pedestal [38]. Notably, the putative Cys-His active site in Ibe contains an aspartatic acid residue in place of the histidine (Fig. 3A). This substitution suggests the function of Ibe may be independent of its putative protease activity. Another homolog, OspB from *S. flexneri*, was shown to share several traits with the effector OspF that inhibits the MAPK pathway [19], but another study found OspB activates the NFκB pathway in a GEF-H1 dependent manner [20]. While VPA1380 was toxic to yeast in non-stress conditions, OspB was shown to only reduce yeast growth when yeast were grown under stress conditions in the presence of caffeine, a purine analog that elicits pleiotropic effects in yeast. [24]. VPA1380’s toxicity was exacerbated by osmotic stress which is toxic to yeast with disruptions in ion homeostasis or the MAPK equivalent HOG (High Osmolarity Glycerol) pathway [22]. Therefore, VPA1380 may target a different eukaryotic pathway than OspB that is required for growth under normal conditions (in the absence of stress) and osmotic stress.

As pathogenic strains of *V. parahaemolyticus* become more prevalent around the world, it is important to understand the bacteria’s virulence mechanisms for diagnostic and treatment purposes. The discovery of VPA1380 as a T3SS2 effector adds a new component to the wide repertoire of *V. parahaemolyticus* virulence factors. By understanding VPA1380’s similarities to the CPD of MARTX and CGT toxins, we are closer to understanding the function of VPA1380 and identifying its cellular target. Future studies will provide insight on how VPA1380 and its close homologs contribute to the infection process of *V. parahaemolyticus* and other major pathogens, and whether it plays a role in the T3SS2-mediated host cell invasion of *V. parahaemolyticus*.

## Materials and Methods

### Strains and growth conditions

The *V. parahaemolyticus* strains POR1(Δ*tdha/s*), POR2(POR1Δ*vcrd1*), and POR2(POR1Δ*vcrd2*) were all derived from the RIMD 2210633 parental strain, which was generously donated by Dr. Tetsuya Iida and Dr. Takeshi Honda [21]. *V. parahaemolyticus* strains were grown in LB media supplemented with NaCl to a final concentration of 3% (MLB), and selection for the pBAD/Myc-His vector (Invitrogen) was maintained with 200 µg/µl kanamycin. For routine cloning, the *Escherichia coli* DH5α strain was grown in 2xYT broth at 37°C. The *Saccharomyces cerevisiae* yeast strain BY4741 (MATa his3Δ0 leu2Δ0 met15Δ0 ura3Δ0) was grown in SD media as described [39] at 30°C. The yeast strain BY4741Δ*ipk1* was obtained from a yeast knock out library (OpenBioSystems). HeLa cells (ATCC) were cultured in DMEM (Invitrogen) supplemented with 10% heat-inactivated fetal bovine serum (Sigma) at 37°C in 5% CO_2_.

### Plasmids

For the *vpa1380-cyaA* fusion construct, the *vpa1380* gene, without its stop codon, and including its −1 kb region were PCR amplified from POR1 genomic DNA and cloned into pBad/Myc-His with the SacI and PstI restriction sites. The catalytic region of the adenylate cyclase gene, *cyaA*, from *Bordetella pertussis* was cloned into the same vector with the PstI and EcoRI restriction sites with a stop codon in the primer since only a partial region of *cyaA* was cloned. For galactose-inducible expression of VPA1380 in yeast, the *vpa1380* gene was cloned into pDGFP [40] with the XbaI and SacI restriction sites, in-frame with a C-terminal eGFP-myc tag. Site-specific mutagenesis was performed using the QuikChange Kit (Statagene) according to the manufacturers instructions. The yeast *ipk1* gene was PCR amplified from yeast genomic DNA of the BY4741 strain and cloned into pAML10 (RIKEN) downstream of the ADH1 constitutive promoter with the XbaI and SacI restriction sites.

### Secretion Assay

*V. parahaemolyticus* strains were grown overnight and diluted to OD_600_ = 0.3 in LB supplemented with 0.05% bile salts. Cultures were induced for 3 hours at 37°C with constant agitation. For the lysate fraction, 0.5 OD_600_ of sample was pelleted and resuspended in 2x Laemmli sample buffer. To collect the media fraction, the protein was pelleted with trichloroacetic acid (10% v/v) and deoxycholate (150 µg/ml) overnight at 4°C. The precipitated protein was washed with acetone and resuspended in 2x Laemmli sample buffer. Protein was detected with the mouse anti-CyaA antibody (Invitrogen).

### Translocation assay

*V. parahaemolyticus* strains were grown overnight and diluted to OD_600_ = 0.3 in LB supplemented with 0.05% bile salts. Cultures were pre-induced for 1 hour at 37°C and then added at an MOI of 10 to confluent HeLa cells in a 96 well plate. The cells were spun down at 1000 g for 5 minutes and then incubated for 1 hour at 37°C. Intracellular cAMP levels were measured with the cAMP Direct Biotrak EIA kit (GE Healthcare Life Sciences) according to the manufacture’s directions.

### Bioinformatics

VPA1380-related sequences were collected using default settings of BLAST [41] against the NR database with the sequence query (GI|28901235|). To identify the closest related structures, we queried the PDB70 database (April 10, 2014) with the same sequence using the HHPRED server [42]. VPA1380-related sequences were aligned to the top identified structure sequences (3fzy A, 3pa8A, and 3ho6A) using PROMALS3D [43], with manual adjustments guided by secondary structure predictions for VPA1380 (JPRED server [44]), HHPRED alignments, and conserved hydrophobicity patterns. The resulting alignment extended the initial HHPRED alignment to include the VPA1380 C-terminus, but is lacking a portion of the N-terminus (starts at residue 116).

### Yeast growth assays

Spotting assays were performed as described [39]. Briefly, BY4741 strains were grown overnight in SD media. Yeast were then normalized to OD_600_ = 1.0 and 5-fold dilutions were spotted (10 µl) on SD agar plates under non-inducing conditions (2% glucose) or inducing conditions (2% galactose and 1% raffinose) with and without 0.5 M NaCl. Production of effector protein was detected with mouse anti-GFP (Clontech) antibody as previously described [39].

## Supporting Information

Figure S1 **VPA1380 is homologous to OspB from** ***Shigella flexneri.*** Sequence homology of VPA1380 and *Shigella flexneri* effector OspB. The (*) represents identical amino acids, (:) represents conservative substitutions, and (.) represents semiconservative substitutions.
(TIF)

Figure S2 **VPA1380’s CPD is sufficient for toxicity in yeast. (A)** Growth of yeast expressing VPA1380Δ50-eGFP, which encodes the CPD region, and putative active site mutant. 10-fold serial dilutions of yeast were spotted on repressing (glucose) or inducing (galactose) medium. **(B)** Detection of eGFP, VPA1380Δ50-eGFP, and VPA1380Δ50-eGFP by immunoblot analysis. Blots were probed with anti-GFP antibody.
(TIF)

Figure S3 **Verifying protein production in yeast spotting assay examining VPA1380’s putative catalytic residues.** Detection of eGFP, VPA1380-eGFP, and VPA1380-eGFP point mutants by immunoblot analysis from strains examined in Figure 2. Blots were probed with anti-GFP antibody.
(TIF)

Figure S4 **Verifying protein production in yeast spotting assays examining VPA1380’s dependence on IP6.** Detection of eGFP, VPA1380-eGFP, and VPA1380-eGFP point mutants by immunoblot analysis from strains examined in **(A)** Figure 4A and **(B)** Figure 4B. Blots were probed with anti-GFP antibody.
(TIF)
