# Supplementary figures and images for: Vibrio Type III Effector VPA1380 Is Related to the Cysteine Protease Domain of Large Bacterial Toxins
Source: PLoS One. 2014 Aug 6;9(8):e104387. doi: 10.1371/journal.pone.0104387 (PMC4123922; doi:10.1371/journal.pone.0104387)

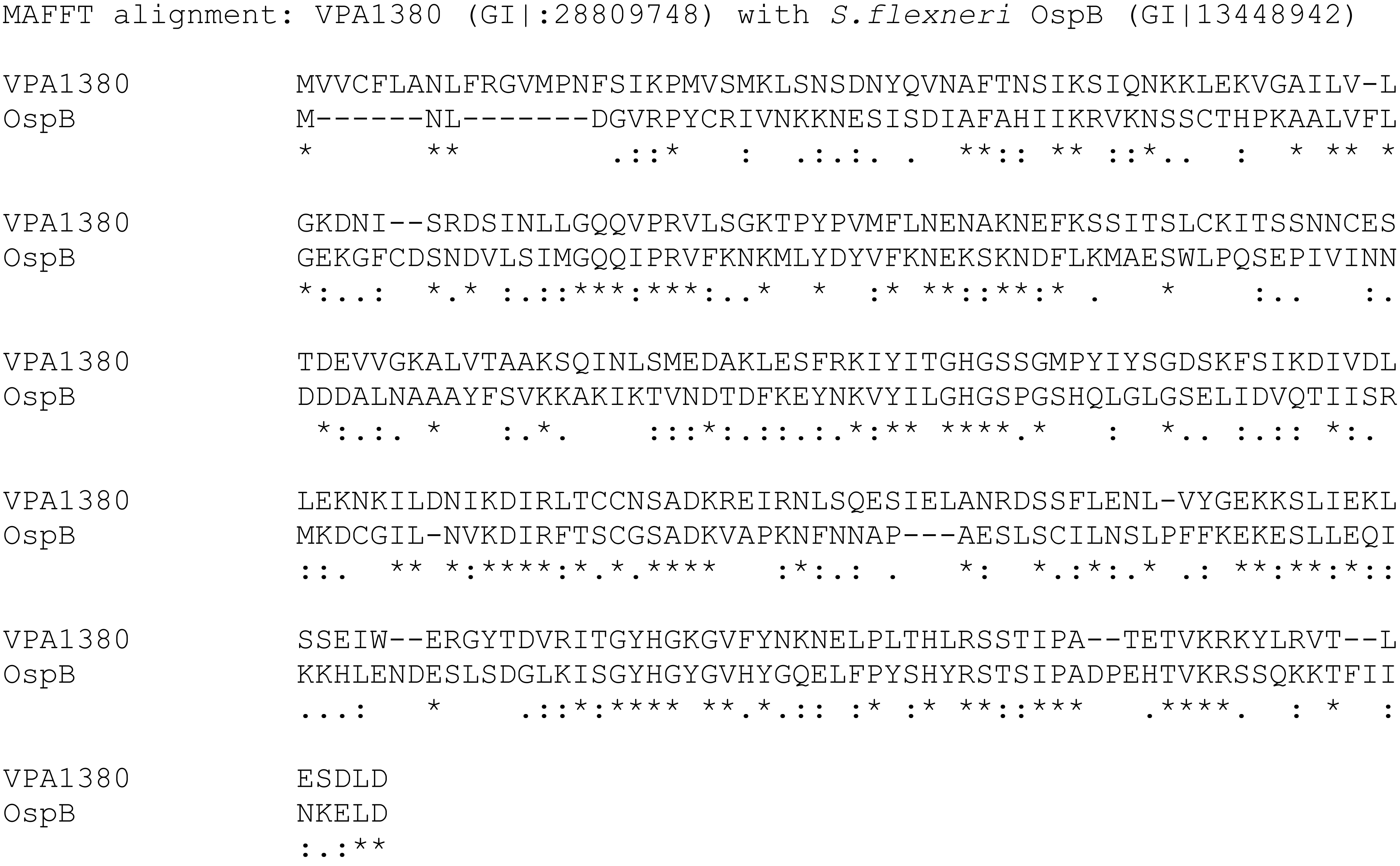

Supplement: Figure S1 — VPA1380 is homologous to OspB from Shigella flexneri. Sequence homology of VPA1380 and Shigella flexneri effector OspB. The (*) represents identical amino acids, (:) represents conservative substitutions, and (.) represents semiconservative substitutions. (TIF) [file pone.0104387.s001.tif]

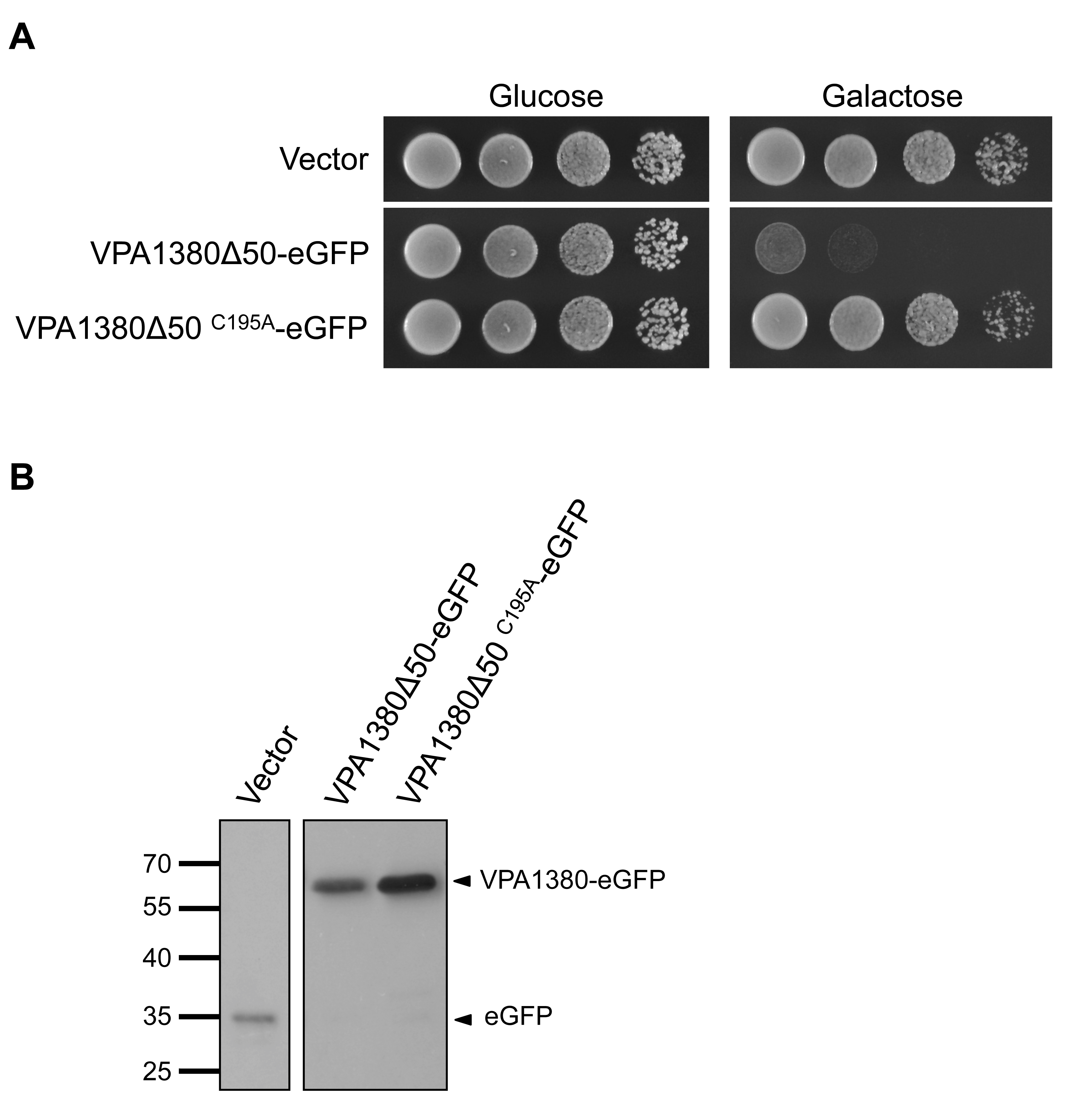

Supplement: Figure S2 — VPA1380’s CPD is sufficient for toxicity in yeast. (A) Growth of yeast expressing VPA1380Δ50-eGFP, which encodes the CPD region, and putative active site mutant. 10-fold serial dilutions of yeast were spotted on repressing (glucose) or inducing (galactose) medium. (B) Detection of eGFP, VPA1380Δ50-eGFP, and VPA1380Δ50-eGFP by immunoblot analysis. Blots were probed with anti-GFP antibody. (TIF) [file pone.0104387.s002.tif]

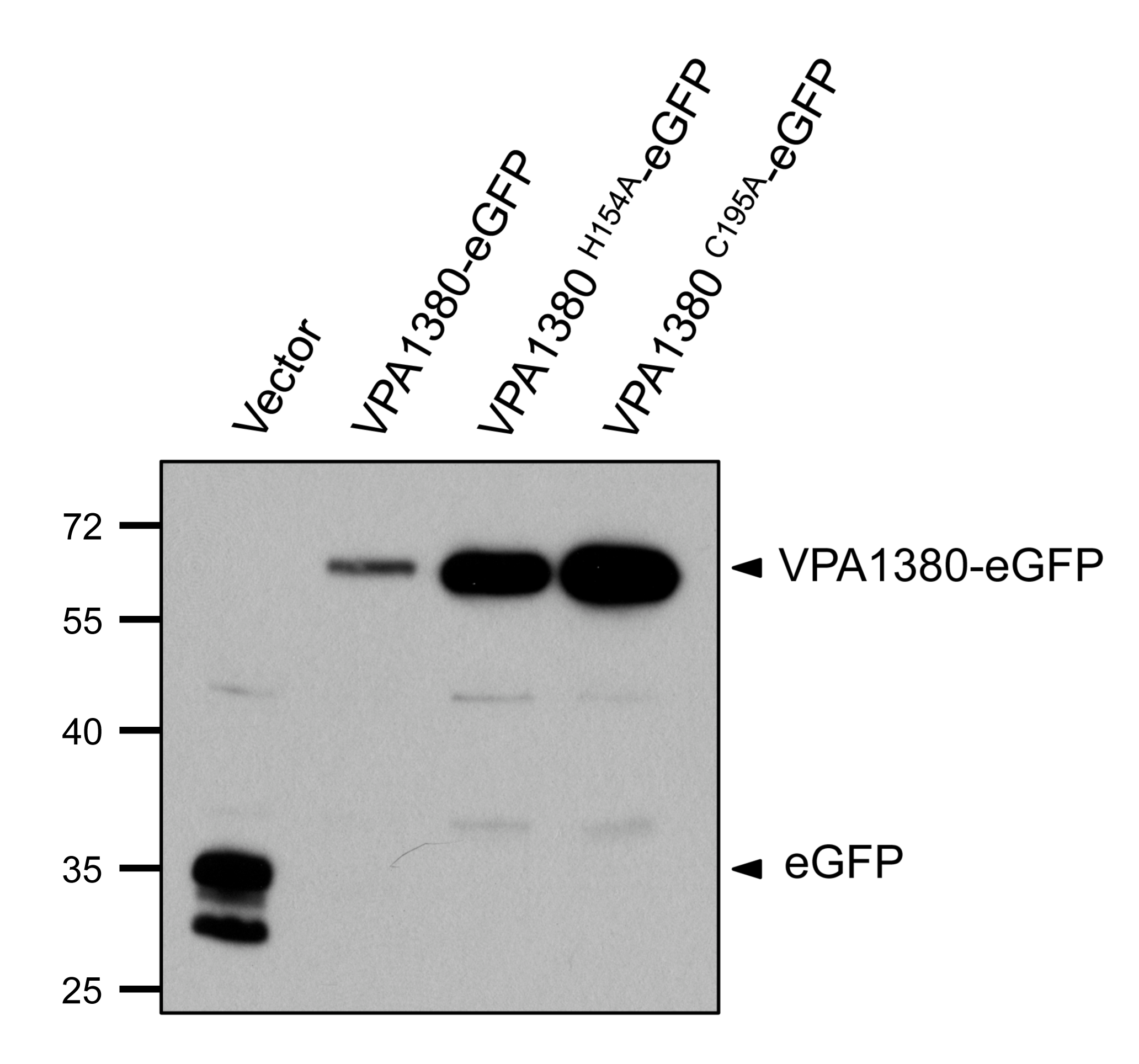

Supplement: Figure S3 — Verifying protein production in yeast spotting assay examining VPA1380’s putative catalytic residues. Detection of eGFP, VPA1380-eGFP, and VPA1380-eGFP point mutants by immunoblot analysis from strains examined in Figure 2. Blots were probed with anti-GFP antibody. (TIF) [file pone.0104387.s003.tif]

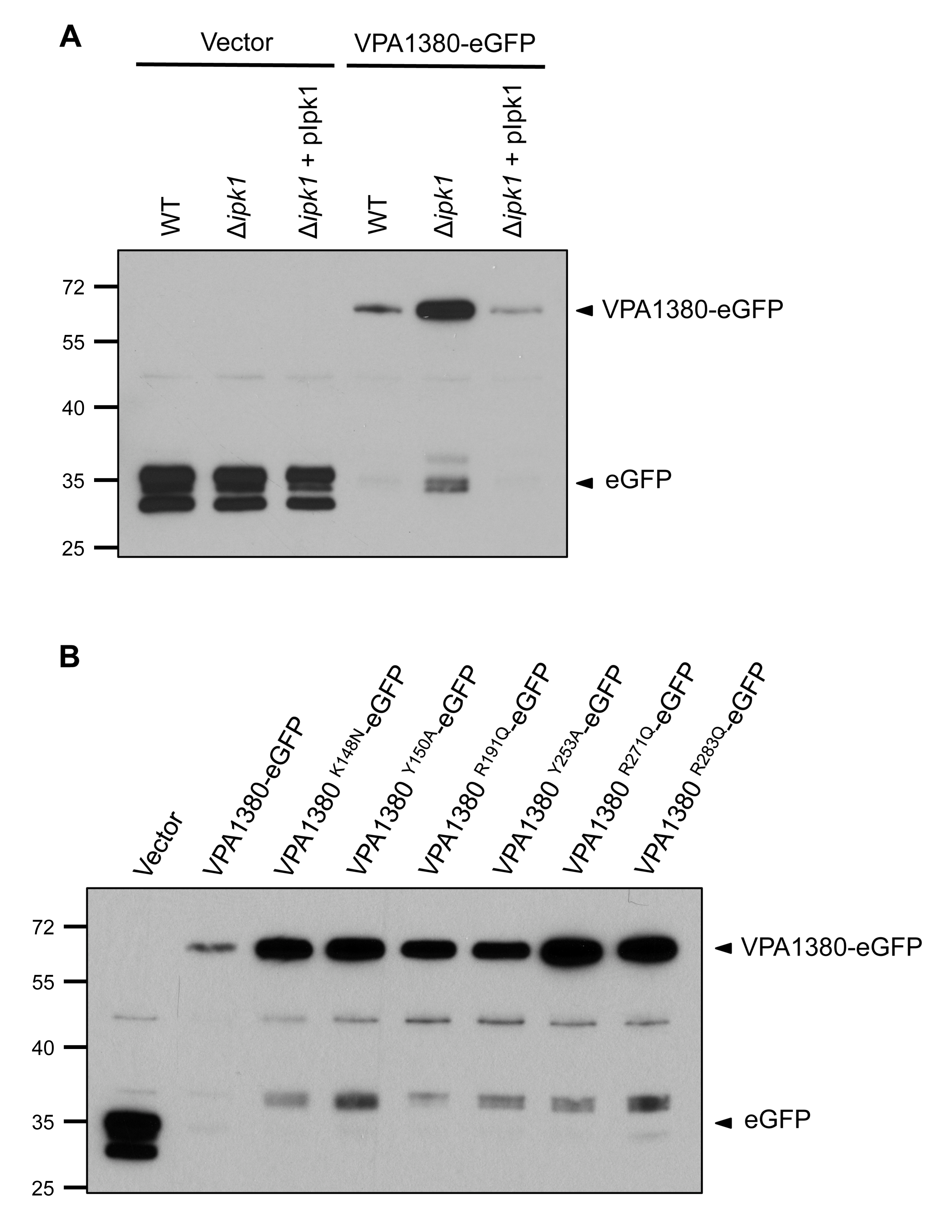

Supplement: Figure S4 — Verifying protein production in yeast spotting assays examining VPA1380’s dependence on IP6. Detection of eGFP, VPA1380-eGFP, and VPA1380-eGFP point mutants by immunoblot analysis from strains examined in (A) Figure 4A and (B) Figure 4B. Blots were probed with anti-GFP antibody. (TIF) [file pone.0104387.s004.tif]
